# Relationship between the presence of baccalaureate-educated RNs and quality of care: a cross-sectional study in Dutch long-term care facilities

**DOI:** 10.1186/s12913-016-1947-8

**Published:** 2017-01-19

**Authors:** Ramona Backhaus, Erik van Rossum, Hilde Verbeek, Ruud J. G. Halfens, Frans E. S. Tan, Elizabeth Capezuti, Jan P. H. Hamers

**Affiliations:** 1 Department of Health Services Research, Maastricht University, CAPHRI Care and Public Health Research Institute, P.O. Box 616, 6200 MD Maastricht, The Netherlands; 2 Zuyd University of Applied Sciences, Research Centre on Autonomy and Participation, P.O. Box 550, 6400 AN Heerlen, The Netherlands; 3 Department of Methodology and Statistics, Maastricht University, CAPHRI Care and Public Health Research Institute, P.O. Box 616, 6200 MD Maastricht, The Netherlands; 4 Hunter College, City University of New York, Brookdale Campus West, Room 526, 425 E. 25th Street # 925, New York, NY 10010 USA

**Keywords:** Quality of care, Registered nurses, Nursing homes, Long-term care facilities, Staffing

## Abstract

**Background:**

Recent evidence suggests that an increase in baccalaureate-educated registered nurses (BRNs) leads to better quality of care in hospitals. For geriatric long-term care facilities such as nursing homes, this relationship is less clear. Most studies assessing the relationship between nurse staffing and quality of care in long-term care facilities are US-based, and only a few have focused on the unique contribution of registered nurses. In this study, we focus on BRNs, as they are expected to serve as role models and change agents, while little is known about their unique contribution to quality of care in long-term care facilities.

**Methods:**

We conducted a cross-sectional study among 282 wards and 6,145 residents from 95 Dutch long-term care facilities. The relationship between the presence of BRNs in wards and quality of care was assessed, controlling for background characteristics, i.e. ward size, and residents’ age, gender, length of stay, comorbidities, and care dependency status. Multilevel logistic regression analyses, using a generalized estimating equation approach, were performed.

**Results:**

57% of the wards employed BRNs. In these wards, the BRNs delivered on average 4.8 min of care per resident per day. Among residents living in somatic wards that employed BRNs, the probability of experiencing a fall (odds ratio 1.44; 95% CI 1.06-1.96) and receiving antipsychotic drugs (odds ratio 2.15; 95% CI 1.66-2.78) was higher, whereas the probability of having an indwelling urinary catheter was lower (odds ratio 0.70; 95% CI 0.53-0.91). Among residents living in psychogeriatric wards that employed BRNs, the probability of experiencing a medication incident was lower (odds ratio 0.68; 95% CI 0.49-0.95). For residents from both ward types, the probability of suffering from nosocomial pressure ulcers did not significantly differ for residents in wards employing BRNs.

**Conclusions:**

In wards that employed BRNs, their mean amount of time spent per resident was low, while quality of care on most wards was acceptable. No consistent evidence was found for a relationship between the presence of BRNs in wards and quality of care outcomes, controlling for background characteristics. Future studies should consider the mediating and moderating role of staffing-related work processes and ward environment characteristics on quality of care.

## Background

Recent evidence suggests that higher staffing levels and an increase in baccalaureate-educated registered nurses (BRNs) lead to better quality of care (QoC) in hospitals [1]. For long-term care facilities (LTCFs) such as nursing homes, this relationship is less clear [2, 3]. It is assumed that an increase in BRNs could lead to an improvement in quality of life and QoC for LTCF residents as well. However, in most countries, the number of BRNs in LTCFs is low [4]. Traditionally, working in LTCFs is associated with a low status career and inadequate salaries [5], reducing the chance to attract sufficient BRNs. When present BRNs currently often fulfill management positions. If involved in daily care, they frequently perform similar tasks as less educated staff. Their unique expertise could be used to serve as a role model, supervisor or innovator in the facility. As the number of less educated staff in LTCFs is high, BRNs can advance other staff practice to improve QoC [2, 4, 6]. The importance of BRNs in LTCFs, and especially in nursing homes, is expected to increase further as new models of care will likely be implemented in the near future that require high level coordination and evaluation skills [7], and BRNs are expected to have more of these abstract thinking skills than less educated staff [8].

International evidence for the added value of BRNs in LTCFs is scarce [2, 3]. Most studies assessing the relationship between nurse staffing and QoC in LTCFs are US-based [2, 3, 9], and only a few focus on the unique contribution of RNs [4, 10]. Most authors do not clarify the educational level of RNs, even though their educational backgrounds may differ substantially [11]. This study focuses on the unique contribution of BRNs in LTCFs. The aim of this study was to examine the relationship between the presence of BRNs in wards and QoC in Dutch LTCFs. As a national database on staffing and QoC is lacking in the Netherlands [12], we conducted this study in cooperation with the Dutch Prevalence Measurement of Care Problems (LPZ: Landelijke Prevalentiemeting Zorgproblemen) [13]. The LPZ measurement is an annual, multicenter, cross-sectional point prevalence measurement of several care problems in LTCFs (such as pressure ulcers and fall incidents).

In this study, we focus on nurse sensitive indicators of QoC. The relationship between the presence of BRNs in wards and outcomes that are most sensitive to nursing care is addressed. We chose the following five outcomes from the LPZ database: nosocomial pressure ulcers, medication incidents, falls, antipsychotic drug use, and urinary indwelling catheter use. Pressure ulcers are the most frequently used QoC outcome for assessing the relationship between nurse staffing and QoC in LTCFs and seem to be a nurse-sensitive outcome [2, 3]. Ideally, only nosocomial pressure ulcers, which are pressure ulcers that developed during a resident’s stay in the LTCF, should be considered.

In previous studies, higher nurse staffing levels in LTCFs were associated with a decrease in falls [14–17], but evidence on the relationship between better educated staff and the occurrence of falls in LTCFs is lacking. In addition, evidence is absent for a relationship between the presence of RNs in wards and medication incidents in LTCFs. However, we expect that medication incidents can be seen as a nurse-sensitive outcome as RNs spend much time on medication-related activities [18, 19]. Nevertheless, the occurrence of falls or medication incidents in LTCFs should be prevented as both can have serious consequences for residents, e.g. fall-related injuries or adverse drug events.

The prevalence rates of antipsychotic drug use in LTCFs are often high [20]. We assume that the high prevalence rates can partly be explained by the inappropriate use of antipsychotic drugs, associated with poor QoC [10, 20]. Antipsychotic drug use is defined as inappropriate when a clinical rationale is absent such as a diagnosis of delirium, schizophrenia, or psychotic disorder. Recent studies suggest that the prescription of antipsychotics is not based on clinical reasons alone, but that direct care staff in nursing homes often believe that antipsychotics are the only treatment choice to manage challenging resident behaviors including screaming, moaning or wandering [20, 21]. The critical thinking skills of BRNs may place them in a better position to address challenging resident behavior without using antipsychotics, and might lead to less antipsychotic drug use on wards with higher BRN staffing levels.

Previous studies have considered fewer indwelling urinary catheters as a proxy for better urinary incontinence status of nursing home residents [2, 22, 23], and showed that more RN staff was associated with fewer catheterizations [22, 23]. The use of urinary indwelling catheters should be prevented as they can cause urinary tract infections, resident discomfort, and decreased mobility [10, 24]. BRNs are expected to have a better understanding of these negative consequences. Therefore, the prevalence rate of residents with indwelling urinary catheters might be lower on wards where BRNs are present.

## Methods

### Study design

This study was conducted in cooperation with the Dutch LPZ cross-sectional point prevalence measurement in April 2014. Annually, the LPZ measurement takes place on the same day in different health care settings. Participation of health care organizations is voluntary [13]. Data are collected at the organizational, ward, and resident level, using standardized questionnaires. Each participating organization appoints one coordinator who collects data at the organizational level, whereas ward managers provide data on their specific ward. Resident data (resident characteristics and prevalence of QoC outcomes) are collected by two health care professionals, one working on the resident’s ward and one from another ward [13]. Inter-rater reliability between observers was found to be good (Cohen’s kappa 0.87) [13, 25, 26]. The standardized questionnaires are based on psychometrically tested instruments, existing guidelines or literature reviews, and are developed and regularly updated in collaboration with experts [27–34].

To obtain BRN staffing data, we added 3 questions to the LPZ ward-level questionnaire. For each ward, the total number of hours of care delivered by BRNs was ascertained, as well as time spent in direct resident care (personal and nursing care, e.g. help with activities of daily living) and indirect care (e.g. staff education, coaching, and care innovation projects). No data were available on total nurse staffing.

### Setting and participants

In Dutch LTCFs, most wards provide complex nursing care, whereas some wards provide only assistance with domestic tasks [35]. Typically, long-term nursing care for older adults in the Netherlands is provided in somatic (for residents with physical disabilities) and psychogeriatric (for residents with dementia) wards [36]. Therefore, we included only residents aged > 60 years from psychogeriatric or somatic nursing care wards.

In the Netherlands, specifically trained nursing home medical specialists provide medical care for LTCF residents [36]. Both these specialists as well as associated health professionals (e.g. psychologists, physiotherapists) are employed by the LTCF. Similar to other countries, the educational level of nursing staff varies. The largest proportion of nursing staff consist of certified nurse assistants (educational level 3) with 2–3 years of vocational training [36]. Dutch certified nurse assistants are comparable to licensed practical/vocational nurses in the United States [37]. There are also nurse assistants (educational level 2), nurse aides (educational level 1) as well as some uneducated staff [38]. In many LTCFs, the lowest percentage of staff are RNs (educational level 4) and BRNs (educational level 5).

In total, 282 wards and 6,145 residents from 95 LTCFs were included in our study. The 95 LTCFs are managed by 20 Dutch elderly care organizations.

### Data source, variables and measurement

Table 1 presents the study variables and their measurement.

**Table 1:** Study variables and their measurement

Variable	Measurement
Resident characteristics	
Gender	Man/woman
Age	Age in years
Length of stay	Number of days
Comorbidities	Number of comorbidities (0-24^a^): Infectious illness; cancer; endocrine, nutritional or metabolic illness/disease; diabetes mellitus; disease of blood or blood related organs; psychological disorders; dementia; nervous system disorder (excluding cerebrovascular accident (CVA)); spinal cord lesion/paraplegia; cardio vascular disease; CVA/hemiparesis; respiratory disorder/diseases, including nose and tonsils; disorder/disease of the digestive tract, including intestinal obstruction, peritonitis, hernia, liver, gallbladder, pancreas; disorder/disease of kidney/urinary tract, sexual organs; skin disorder/disease; motor disorder/disease; congenital disorders; injury resulting from accident (s), undesirable consequences of accident (s); symptoms and abnormal clinical or lab findings, not elsewhere classified; overdose/substance abuse/addiction; disease of the eye; disease of the ear; pregnancy, child birth; external factors for disease
Care dependency	Care Dependency Scale [39]: For each of the following 15 activities, the degree to which the resident is dependent upon care provided by others is indicated on a 5-point scale (completely dependent (1) – completely independent (5)^a^): eating and drinking, incontinence, body posture, mobility, day/night pattern, getting dressed and undressed, body temperature, hygiene, avoiding danger, communication, contact with others, sense of rules and values, daily activities, recreational activities, learning ability [13]. For each resident, the total score (sum of 15 items) was divided by 15 to obtain a mean score.
Presence of BRN	
Presence of BRN	At least one BRN present in ward
Quality of care outcomes	
Nosocomial pressure ulcers	Resident suffers from at least one nosocomial pressure ulcer category 2–4 (European Pressure Ulcer Advisory Panel (EPUAP) & National Pressure Ulcer Advisory Panel (NPUAP) [13, 41]): - Category 2: Partial thickness - Category 3: Full thickness skin loss - Category 4: Full thickness tissue loss
Medication incidents	Resident had at least one medication incident during the last 30 days ^b^: - Omitted dose
	- Wrong dose
	- Wrong time taken
	- Wrong drug
	- Wrong drug administration
Falls	Resident has fallen at least once during last 30 days ^b^
Antipsychotic drug use	Antipsychotic drug use during last 7 days ^b^
Indwelling urinary catheter use	Resident has an indwelling urinary catheter in place at the time
Ward characteristics	
Ward type	Psychogeriatric/somatic nursing care ward
Ward size	Number of residents living on ward

^a^underlined score is the most favorable score

^b^answered by resident or responsible nurse and/or indicated in resident file [13]

#### Resident characteristics and QoC outcomes

Residents’ age, gender, length of stay, number of comorbidities, and care dependency status (CDS) [39] were extracted from the LPZ, as well as the following QoC outcomes that were dichotomized (yes/no): nosocomial pressure ulcers, falls, antipsychotic drugs, medication incidents, and urinary indwelling catheters.

#### Presence of BRN

The total hours of care delivered by BRNs, as well as their hours spent on direct resident care and indirect care practices, were extracted from the LPZ. This data was used to distinguish between wards with at least one BRN present and wards that did not employ BRNs.

#### Ward characteristics

The ward type (somatic or psychogeriatric) as well as the ward size (number of residents living on ward) were extracted from the LPZ.

### Statistical analyses

Data were analyzed with SPSS for Windows (version 22).

#### Missing data

In the Dutch LPZ, each participating organization can decide which QoC outcomes are assessed on the resident level within the organization [30]. Therefore, because of non-participation, data on QoC outcomes were partly missing. In addition, for some residents, data collectors were not able to determine whether or not the resident suffered from a QoC problem, leading to missing data as well. The latter was the case for nosocomial pressure ulcers (*n* = 22; 0.4%), falls (*n* = 53; 0.9%) and antipsychotic drug use (*n* = 28; 0.5%).

In total, among residents living in somatic wards, between 1.5% (falls) and 18.2% (nosocomial pressure ulcers) of data were missing. Among residents living in psychogeriatric wards, the amount of missing data ranged from 0.4% (falls) to 12.8% (nosocomial pressure ulcers). We cannot ignore these missing observations since the reasons for not including these by some organizations is not known. Therefore, three different approaches were taken to handle missing data. First, we performed a complete case analysis, ignoring missing data. Second, a sensitivity analysis was performed, in which all cases with missing data on a dependent variable were considered as “not suffering from the disease” (e.g., not having nosocomial pressure ulcers). Third, missing data were imputed, using multiple imputation techniques. To ensure the variability of predictors [40], the imputations were based on 7 (categorical) variables from the data set (BRNs working on ward, ward size, as well as residents’ length of stay, age, gender, number of comorbidities, and care dependency). After performing the sensitivity analyses and the multiple imputations, the findings of these analyses were compared with those from the complete-case analyses.

#### Univariate descriptive statistics

Univariate descriptive statistics were computed. Means and standard deviations were calculated for resident characteristics and BRN staffing. For QoC outcomes, percentages of residents suffering from the outcome were calculated (frequency distribution).

#### (Multilevel) logistic regression analyses

For each QoC outcome, we estimated the relationship between the presence of BRNs in wards and QoC controlling for background characteristics, i.e. ward size, and residents’ age, gender, length of stay, number of comorbidities, and care dependency status.

As the average time spent by BRNs per resident was low, we chose to dichotomize the BRN staffing variable, i.e. *BRN not working on ward* and *BRN working on ward*. Five control variables were recoded into categorical variables to avoid sparse cells and for the ease of interpretation [42]. Ward size was recoded into 4 categories, i.e. *fewer than 12 residents*, *13*–*24 residents*, *25*–*36 residents*, *more than 37 residents*. Age was recoded into 4 categories, i.e. *age 61–70*, *age 71–80*, *age 81–90*, and *age 91–110*. Length of stay was recoded into 6 categories, i.e. *0*–*1 years*, *1*–*2 years*, *2*–*3 years*, *3*–*4 years*, *4*–*5 years*, and *longer than 5 years*. The number of comorbidities was recoded into 5 categories: *1 comorbidity*, *2 comorbidities*, *3 comorbidities*, *4 comorbidities*, and *5 or more comorbidities*. The total CDS score of each resident was changed into 1 of 5 categories (*completely dependent (1) – completely independent (5)*).

Due to differences in the care provided in somatic and psychogeriatric wards, separate analyses were performed among residents living in somatic and psychogeriatric wards. Ideally, to take into account possible correlations between residents living in the same ward and/or LTCF, 3-level logistic regression analyses should have been conducted in which residents were nested in wards and wards were nested in LTCFs. However, as some LTCFs were included with only one ward, it was not possible to conduct 3-level analyses examining the possible impact of wards and LTCFs simultaneously. These analyses led to estimation problems. Alternatively, two different 2-level logistic regression analyses were performed using a generalized estimating equation (GEE) approach. In these multilevel analyses, residents (level 1) were nested in wards (level 2) or residents (level 1) were nested in LTCFs (level 2). To test the correlation within residents living in the same ward or in the same LTCF, the intraclass correlation coefficient (ICC) was considered. Additionally, for each QoC outcome, a general logistic regression analysis was conducted for the resident level, not taking into account any hierarchy of data.

### Ethical considerations

All data were extracted from an existing database (LPZ), in which we received permission to conduct secondary analyses. The LPZ received ethical approval from the Medical Ethics Review Committee (METC) of the University Hospital Maastricht and Maastricht University.

## Results

### Univariate descriptive statistics

#### Ward and resident characteristics

From the 282 participating wards, 117 were somatic wards (2,604 residents) and 165 were psychogeriatric wards (3,541 residents). Resident’s mean age was 84 years (SD ± 8) and 73% of the residents were female. Their mean length of stay was 2.9 years (1057 days (SD ± 1055)), and on average, residents had 3 comorbidities (SD ± 1). The mean CDS was 2.4 (SD ± 1.2), meaning that, on average, residents were functionally dependent.

#### Presence of BRN

57% of the wards employed a BRN, who delivered, on average, 4.8 min of care per resident per day (0.08 nurse hours per resident per day (NHPRD), SD ± 0.08). The BRN conducted direct care practices on 91% of the wards that employed a BRN, and indirect care practices on 80% of the wards. On wards where the BRN had direct care practices, the average time spent on these practices was 3.6 min per resident per day (0.06 NHPRD, SD ± 0.07). On wards where the BRN had indirect care practices, the average time spent on these practices was 1.2 min per resident per day (0.02 NHPRD, SD ± 0.02).

#### QoC

From the residents that participated in our study, on average, 2.6% suffered from nosocomial pressure ulcers (category 2–4), 10.4% had experienced a fall, and 5.3% a medication incident. 7.2% of the residents had an indwelling urinary catheter and 19.6% received antipsychotic drugs. Table 2 shows a considerable variation in prevalence rates among residents between somatic (more likely to have a nosocomial pressure ulcer, medication incident or indwelling urinary catheter) and psychogeriatric wards (more likely to fall or use antipsychotic drugs). When analyzing the relationship between the presence of BRNs in wards and nosocomial pressure ulcers among residents living on psychogeriatric wards, residents who were completely independent (i.e., *CDS 5; n* = 92) were excluded, as none of these residents suffered from nosocomial pressure ulcers.

**Table 2:** Differences in resident characteristics and prevalence rates of quality of care outcomes among residents living in somatic and psychogeriatric wards

	Residents living in somatic wards (*n* = 2604)	Residents living in psychogeriatric wards (*n* = 3541)
Resident characteristics		
Age in years (mean, SD)^a^	83 ± 9	84 ± 7
Female (%)^a^	70	75
Length of stay in years and days (mean, SD)^a^	3.1 (1132 ± 1200)	2.7 (1002 ± 930)
Number of comorbidities (mean, SD)^a^	3 ± 1	3 ± 1
Care dependency (mean, SD)^a^ ^b^	2.9 ± 1.2	2.1 ± 1.1
Quality of care outcomes		
Nosocomial pressure ulcers (%)^a^	3.4 (*n* = 2131)	1.9 (*n* = 3086)
Medication incidents (%)^a^	6.2 (*n* = 2307)	4.6 (*n* = 3451)
Falls (%)^a^	7.6 (*n* = 2564)	12.4 (*n* = 3528)
Antipsychotic drug use (%)^a^	15.2 (*n* = 2296)	22.6 (*n* = 3434)
Indwelling urinary catheter use (%)^a^	11.7 (*n* = 2271)	3.9 (*n* = 3143)

*Note:*

*SD* standard deviation

^a^significantly different among residents living in somatic and psychogeriatric wards (*p* < .01; independent samples *t*-test or chi-square)

^b^degree to which the resident is dependent upon care provided by others is indicated on a 5-point scale (completely dependent (1) – completely independent (5))

### (Multilevel) logistic regression analyses

For each QoC outcome, the results of the multilevel and the general logistic regression analyses were almost identical, and the ICC was low. In addition, the results of complete case analyses and those from the sensitivity analyses, as well as the analyses with imputed data were almost identical. Therefore, we present only the results of the general logistic regression analyses for complete cases (Table 3).

**Table 3:** Associations between presence of BRNs and quality of care indicators^a^

Outcome measure	Ward type	OR (BRN on ward vs. no BRN on ward)	95% CI	*p*-value
Nosocomial pressure ulcers	Somatic	0.68	0.42 - 1.10	.12
	Psychogeriatric	0.79	0.46 - 1.38	.41
Medication incidents	Somatic	1.17	0.82 - 1.67	.39
	Psychogeriatric	0.68	0.49 - 0.95	.02
Falls	Somatic	1.44	1.06 – 1.96	.02
	Psychogeriatric	1.10	0.89 - 1.36	.38
Antipsychotic drug use	Somatic	2.15	1.66 - 2.78	.00
	Psychogeriatric	1.06	0.89 - 1.26	.51
Urinary indwelling catheter use	Somatic	0.70	0.53 - 0.91	.01
	Psychogeriatric	0.96	0.64 - 1.43	.83

^a^Fully adjusted models estimating the relationship between the presence of BRNs and quality of care controlling for background characteristics, i.e. ward size, and residents’ age, gender, length of stay, number of comorbidities, and care dependency status

*Note:*

*BRNs* baccalaureate-educated registered nurses

*OR* odds ratio

*95% CI* 95% confidence interval around OR

As indicated in Table 3, among residents living in somatic wards that employed BRNs, the probability of experiencing a fall (odds ratio 1.44; 95% CI 1.06-1.96) and receiving antipsychotic drugs (odds ratio 2.15; 95% CI 1.66-2.78) was higher, whereas the probability of having an indwelling urinary catheter was lower (odds ratio 0.70; 95% CI 0.53-0.91). Among residents living in psychogeriatric wards that employed BRNs, the probability of experiencing a medication incident was lower (odds ratio 0.68; 95% CI 0.49-0.95). For residents from both ward types, the probability of suffering from nosocomial pressure ulcers did not significantly differ for residents living in a ward that employed BRNs. In addition, among residents living in somatic wards, the probability of experiencing a medication incident did not significantly differ for residents living in a ward that employed BRNs. Among residents living in psychogeriatric wards, the probability of experiencing a fall, receiving antipsychotic drugs, or having an indwelling urinary catheter did not significantly differ for residents living in a ward that employed BRNs.

## Discussion and conclusions

In our study, there was no consistent relationship found between the presence of BRNs in wards and several QoC indicators, controlling for background characteristics. Among residents living in somatic wards that employed BRNs, an increased probability of experiencing a fall and receiving antipsychotic drugs was found, and a decreased probability of having an indwelling urinary catheter. No significant differences were detected for nosocomial pressure ulcers and medication incidents. For residents living in psychogeriatric wards that employed BRNs a decreased probability of experiencing a medication incident was found, whereas the probability for developing any of the other QoC outcomes did not significantly differ.

Two systematic reviews also reported inconsistent findings on QoC indicators [2, 3]. For this study, there are several factors that need to be taken into consideration. First, only 57% of the wards employed a BRN, who delivered, on average, 4.8 min of care per resident per day. BRN staffing levels may not have been high enough to establish better QoC outcomes. For comparison, in a recent Swiss study among 402 wards from 155 nursing homes, on average 32% of all full-time equivalents (FTEs) per ward were RNs [43]. In a recent US study among nursing homes in Colorado [10], RNs spent on average 36 min of care per resident per day. As with all other studies examining the relationship between RN staffing and QoC, both studies did not indicate the educational background of RNs.

Second, for residents living in both types of wards, the prevalence of QoC problems seems low compared to studies conducted in other countries. However, differences in operationalization and measurement methods have to be considered when comparing prevalence rates to other studies [44], making comparisons difficult [26]. The prevalence of nosocomial pressure ulcers was especially low, which may explain why the probability of suffering from nosocomial pressure ulcers did not significantly differ among residents living in wards that did or did not employ BRNs. For both ward types, antipsychotic drug use was the most prevalent QoC problem, yet the prevalence rate of 19.6% was low compared to prevalence rates in other countries. For example, in a study among Belgian nursing home residents the prevalence rate was 32.9% [45]. Nevertheless, the fact that, in our sample, one resident out of every five was provided with antipsychotic drugs, could be a signal of inappropriate drug use. Only unnecessary antipsychotic drug use should be considered as poor QoC. In this study, we were not able to distinguish between (in)appropriate antipsychotic drug use.

Third, the practices of BRNs working in Dutch LTCFs may not differ from those conducted by other nursing staff, meaning that BRNs are not employed optimally to benefit from their unique contribution to QoC outcomes. It seems that most BRNs are responsible for multiple wards, which is reflected in the low amount of time spent per resident per day. BRNs might only see residents that are in acute, complex care situations (e.g., when a decision whether or not to hospitalize the resident has to be made), instead of looking at each resident’s overall care plan.

The findings of this study should be interpreted carefully. The cross-sectional design provides no information about causality. For example, we cannot say whether the employment of BRNs in somatic wards led to an increased probability of receiving antipsychotic drugs or whether BRNs were employed due to high antipsychotic drug use. As some LTCFs were included with only one ward, it was not possible to conduct 3-level analyses examining the possible impact of wards and LTCFs simultaneously. Moreover, we had to focus on BRNs alone, not taking into consideration the contribution of other nursing staff, nursing home medical specialists and allied professionals working in Dutch LTCFs. In addition, due the low average amount of time BRNs spent on wards, we could only distinguish between wards that did or did not employ BRNs, not taking into consideration the actual amount of time BRNs worked on the wards. To compare BRN staffing among wards, we calculated NHPRDs. However, BRNs may only deliver care to residents with the most complex care problems. In our analyses, we distinguished between residents living in somatic and psychogeriatric wards, while in practice, the difference may not be that clear-cut, e.g., some residents living in somatic wards may suffer from dementia or residents living in psychogeriatric wards from somatic diseases as well. Finally, our analyses were limited to the QoC outcomes measured in the LPZ, while BRNs may influence other outcomes, e.g., outcomes related to quality of life of residents. Despite these limitations, our study is the first that provides insight into the relationship between the presence of BRNs in wards and QoC for Dutch LTCFs. As we made use of an existing data infrastructure (LPZ), the sample size was large (6145 residents), and collected data was of good quality.

Although the Dutch government is making efforts to increase the number of BRNs working in elder care, the number of BRNs working in LTCFs is still low, as in 43% of the wards no BRNs were employed. Even for wards that employed BRNs, the mean amount of time spent per resident was low. For LTCFs it is therefore important to carefully think about how to best allocate BRNs on their wards. In recent years, there has been a call to shift emphasis back to the provision of essential nursing care, e.g., providing physical comfort and psychological support or establishing meaningful encounters between staff and residents [46, 47]. It might be the case that BRNs add particular value to improving essential nursing care, thus future studies should consider this. Recently, David Richards has posed the question whether nursing outcomes might need to be defined in terms of a concept called ‘amalgamation of marginal gains’ [47, 48]. During a hospital visit Richards experienced that small, individual actions by nurses only had marginal impact on his well-being, while in total, all these ‘small actions’ significantly reduced his feelings of discomfort and anxiety. By focusing on isolated components of essential nursing care (e.g., communication), Richards stresses one may miss the ‘power of amalgamation’ [47].

In our study, we focused on the presence of BRNs in wards rather than considering staffing as a ‘multidimensional construct’ [49]. Future studies should also consider the mediating and moderating role of staffing-related work processes and ward environment characteristics. For example, more BRNs in the mix of staff might lead to better teamwork and communication, that could result in better QoC [17, 50]. Other examples of work processes BRNs might have influence on are the coordination of care [51], and the collaboration between nursing staff and nursing home medical specialists or allied health professionals [52]. In addition, BRNs might indirectly add value to QoC in LTCFs by acting as a clinical leader and coach for other nursing staff [53]. Moreover, BRNs might also have an influence on ward environment characteristics like the organizational culture or the team climate, which were associated with better QoC in previous studies [54, 55]. Conducting mixed methods-studies, e.g. by combining direct observations with stakeholder interviews, may help to obtain more information on observable behavior (e.g., interactions with residents or other staff and other ‘small actions’) and unobservable cognitive work of BRNs leading to added value for residents, family members, and staff [53].

## Abbreviations

BRNs: Baccalaureate educated registered nurses
LTCFs: Long-term care facilities
NHPRD: Nurse hours per resident per day
QoC: Quality of care
RNs: Registered nurses
